# Flow of Tunable Elastic Microcapsules through Constrictions

**DOI:** 10.1038/s41598-017-11950-2

**Published:** 2017-09-19

**Authors:** Débora F. do Nascimento, Jorge A. Avendaño, Ana Mehl, Maria J. B. Moura, Marcio S. Carvalho, Wynter J. Duncanson

**Affiliations:** Department of Mechanical Engineering, Pontificia Universidade Catolica do Rio de Janeiro, Rio de Janeiro, RJ Brazil

## Abstract

We design and fabricate elastically tunable monodisperse microcapsules using microfluidics and cross-linkable polydimethylsiloxane (PDMS). The overall stiffness of the microcapsules is governed by both the thickness and cross-link ratio of the polymer shell. Flowing suspensions of microcapsules through constricted spaces leads to transient blockage of fluid flow, thus altering the flow behavior. The ability to tune microcapsule mechanical properties enables the design of elastic microcapsules that can be tailored for desired flow behavior in a broad range of applications such as oil recovery, reactor feeding, red blood cell flow and chemical targeted delivery.

## Introduction

Particle flow through confined spaces is important in many industrial and biomedical applications including, enhanced oil recovery (EOR), reactor feeding, filtration, lubrication, red-blood-cell transport, and drug delivery^1–4^. To design particles for such applications, an understanding of the characteristics of their flow behavior through confined media is required. Generally, particles deform to squeeze through smaller geometries which results in a transient partial blockage of fluid flow, thereby limiting fluid mobility causing an increase in fluid pressure. Upon achieving a critical pressure increase that is dependent on the particle’s mechanical properties, the particle dislodges and continues to flow. This pressure increase is problematic in pneumatic feeding of reactors^3^, yet advantageous for EOR applications because the reduction in fluid mobility leads to flow redistribution in the porous space, resulting in increased volumes of oil recovered. Thus, it is essential to design particles with tunable mechanical properties that can be used to induce a range of fluid pressures as a means to control the flow behavior of suspensions in constricted spaces. However, designing this required range of tunability of mechanical properties into traditional particles such as emulsions and preformed polymer gels (PPG) remains challenging. In such systems, the range of fluid flow mobility achieved by controlling the particle's properties is narrow. In surfactant-stabilized emulsions, the range of interfacial tension is small and cannot be used as an effective control parameter; the dispersed phase viscosity is the only available control variable. Solid particles, such as PPGs, cannot be submitted to very large deformation to flow through constrictions smaller than their diameter and therefore are not effective as a mobility control additive. A liquid core-shelled microparticle such as a polymer microcapsule with multiple adjustable physical parameters, including shell thickness and shell elastic modulus, would provide a wide range of fluid mobility and have increased utility for a broad range of flow conditions and applications.

A microcapsule with known and tunable mechanical properties can be prepared through the use of highly-controlled synthesis techniques such as droplet microfluidics; these enable control of microcapsule size and membrane thickness through careful manipulation of fluid flow rates. In addition, microfluidics, particularly glass capillary microfluidics, affords the flexibility and choice of materials^5^ to impart various mechanical properties. An elastomer such as polydimethylsiloxane (PDMS) is an ideal shell material because it has high elastic deformation^6–9^. In addition, the mechanical properties^10,11^ of the PDMS can be easily tuned by changing the ratio of the two-component PDMS precursor^8^. Despite the potential to tune the stiffness of PDMS microparticles, this parameter remains unvaried for solid PDMS^6,8,12^, gas-filled^5,13,14^ and liquid-filled microcapsules^7,9^.

Single compression confinement methods^5,9,15–17^ are commonly used to determine the overall microparticle stiffness. In addition, the pressure-drop associated with microparticle blockage and passage through confined geometries is measured and correlated to the microparticle properties^5,18–23^. The combination of compression and constriction techniques provides a quantitative measure of the mechanical properties of the microparticles, and the effect of particle stiffness on the flow behavior through confined geometries. Such a well-characterized and elastically tunable PDMS shelled microcapsule could be used to adjust fluid mobility in complex constricted media.

In this paper, we use microfluidics to prepare batches of PDMS-shelled microcapsules. Adjusting the fluid flow rates and the cross-link ratio of the two-component PDMS precursor enables production of PDMS-shelled microparticles with tunable elastic properties. We use single particle compression tests to measure their overall stiffness and flow them through constricted capillaries, thereby evaluating their potential to control fluid mobility through confined geometries.

## Results and Discussion

**Figure 1:**
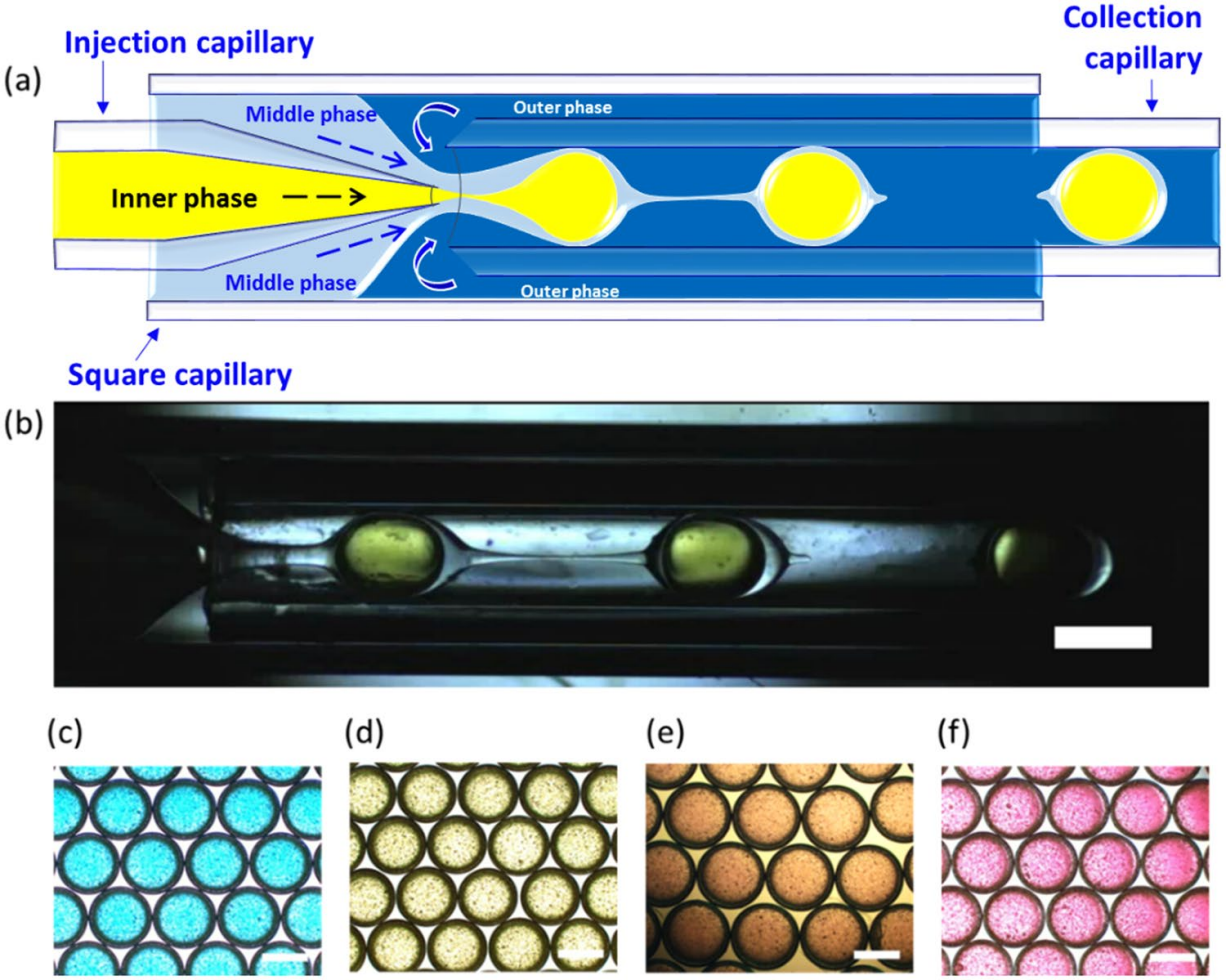
Glass capillary device used to produce monodisperse PDMS microcapsules with tunable elastic properties. (**a**) Sketch of geometry and flow pattern; (**b**) Image of double emulsion production process. Flow focusing by the outer phase causes middle phase filament breakup and drop formation; (**c**–**f**) Images of the monodispersed microcapsules obtained with different polymer to cross-linker ratio (5:1, 10:1, 17.5:1 and 20:1). Each scale bars is 500 μm.

To produce monodisperse PDMS microcapsules, we use a double emulsion glass capillary device^24^, which consists of an outer square capillary, one round injection capillary, and one round collection capillary, as shown in Fig. 1. The tapered injection capillary and the unmodified round collection capillary are inserted from opposite ends into the square capillary and coaxially aligned within the square, as shown schematically in Fig. 1a. The geometry of the double emulsion glass capillary device is held constant while the fluid flow rates are independently varied and controlled through the use of syringe pumps coupled to the device by tubing. The inner water phase comprising of dyed Milli-Q^®^ is pumped through the tapered round capillary, whereas the middle phase, PDMS, co-flows in the space between the square and the round tapered capillary. The outer fluid, a 10% PVA solution, flows in the opposite direction in the space between the square capillary and the blunt-end round capillary to flow-focus the fluids to enable microcapsule formation. A high-speed camera acquiring images at a frame rate of 1000 f/s is used to monitor and capture images of microcapsule production, as shown in Fig. 1b and supplementary video 1. We collect the microcapsules in Milli-Q^®^ water, and rotate them for 1 week to ensure the center droplet and the outer droplet are concentric upon curing at room temperature.

To tune the stiffness of the shells, the PDMS polymer to cross-linker ratio is altered from 5:1, 10:1, 17.5:1 and 20:1. The degree of cross-linking for the pure elastomer is proportional to the elastic modulus: the high-ratio cross-linked PDMS, 5:1, is the most rigid^10,11^. To visually distinguish between the varied cross-linker ratios, the inner water phase is dyed blue for 5:1, yellow for 10:1, orange for 17.5:1 and red dye for 20:1. The capsules from each batch are monodisperse independent of the cross-linker ratio used in droplet fabrication, as seen in Fig. 1c–f.

**Figure 2:**
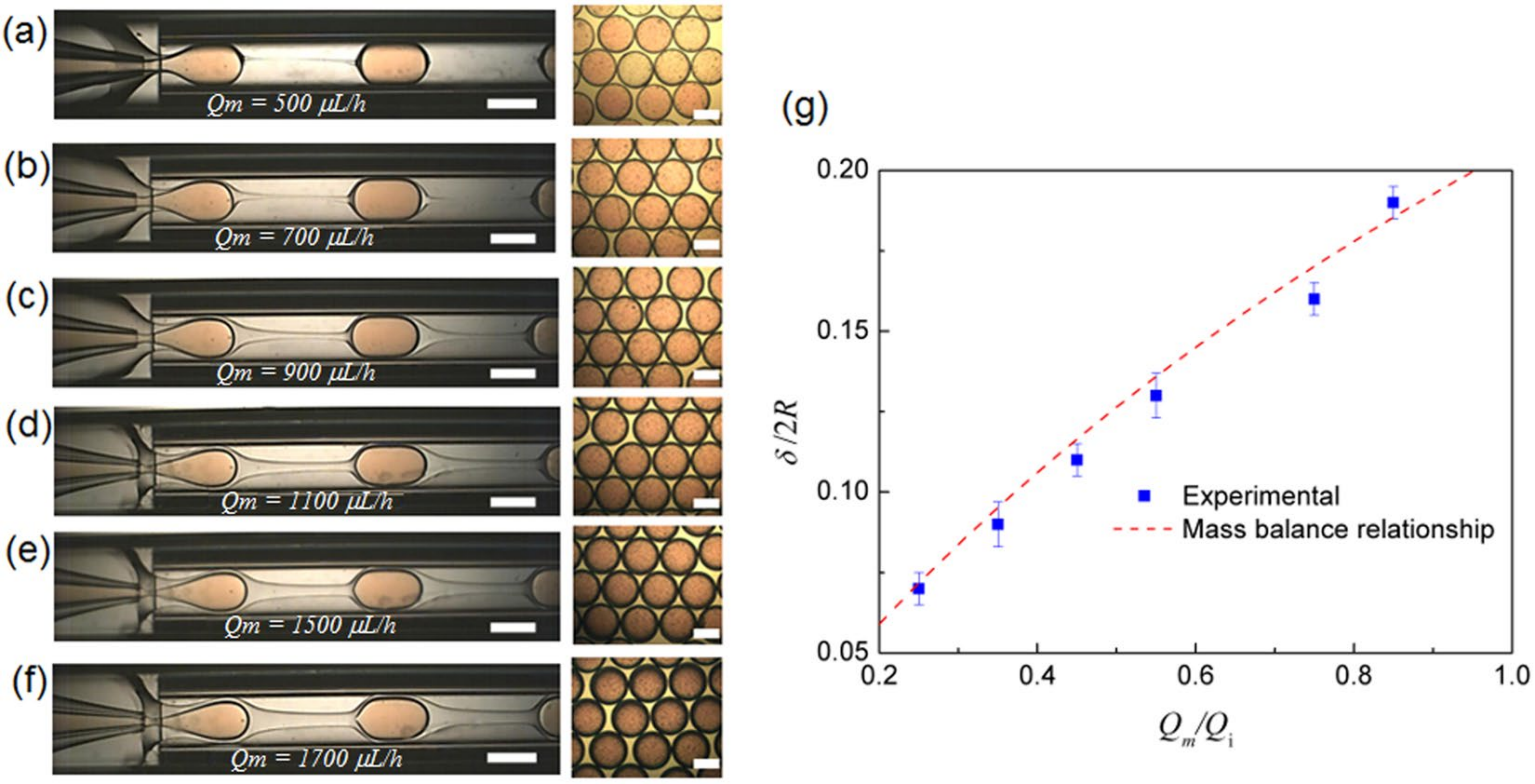
(**a**–**f**) Images of double emulsion production for increasing middle phase flow rate, leading to microcapsules with increasing shell thickness. The capsule production rate is approximately 225 capsules per minute. Each scale bar is 500 μm. (**g**) Shell thickness as a function of middle to inner phase flow rate ratio (*Q*_*m*_ */Q*_*i*_). Dotted line represents the shell thickness predicted by mass balance^9^.

We prepare microcapsules with diameter 2 *R* of approximately 700 µm, with no more than 1.5% deviation from the mean for each batch, and vary shell thicknesses *δ* by changing only the middle phase flow rate (*Q*_*m*_). The flow is initially stabilized at the smallest possible *Q*_*m*_ which renders capsules with the thinnest shell. The thickness of the surrounding middle phase increases with the flow rate *Q*_*m*_; this delays microcapsule breakup and leads to thicker shells, as shown qualitatively in Fig. 2a–f. Remarkably, a simple mass balance^9^ (Equation 1) describes the relationship between the microcapsule thickness and the middle and inner phase flow rate ratio *Q*_*m*_ */Q*_*i*_, as shown in Fig. 2g; this enables *a priori* prediction of flow rates to obtain desired shell thicknesses.

1 $$\frac{\delta }{2R}=1-{(1-\frac{{Q}_{m}}{{Q}_{i}})}^{-\frac{1}{3}}$$

**Figure 3:**
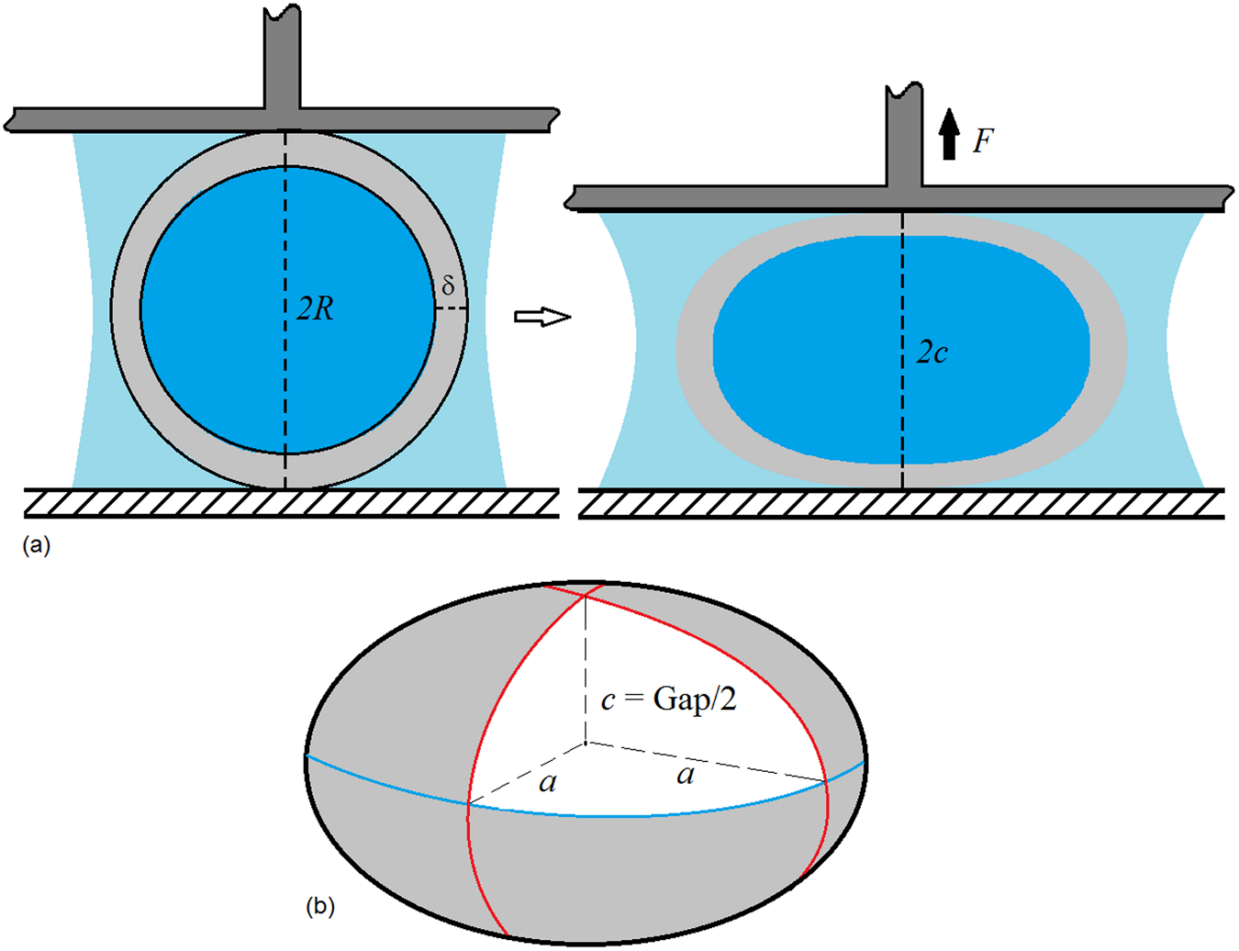
(**a**) Sketch of compression test used to characterize the mechanical response of the produced microcapsules. (**b**) Spherical capsules deform to oblate spheroids with radius *a*.

Our microfluidic technique combined with the use of cross-linkable PDMS elastomer enables us to precisely tune the mechanical properties of the microcapsules. To measure the mechanical properties and to evaluate the degree to which these parameters affect the overall stiffness, we conduct single particle compression tests. For each test, an individual microcapsule is submerged in water between two parallel plates on a rheometer^25^. Initially, the microcapsule is axisymmetrically positioned between the two plates separated at a distance equivalent to the droplet diameter (*2 R*), as shown in Fig. 3a. As the top plate is incrementally lowered, the gap between the plates (*2c*) decreases and the capsule deforms to an oblate spheroid-shape, as shown in Fig. 3b. At each position, the normal force (*F*) is measured until the force reaches steady-state. Assuming constant volume^26^ of the compressed and uncompressed states enables calculation of the radius of the spheroid *a*. The stress *σ* and the shell surface area *A* are evaluated at each plate position as shown in Equations 2 and 3:

2 $$\sigma =(\frac{F}{\pi {a}^{2}});$$

3 $$A=2\pi {a}^{2}[1+(\frac{{c}^{2}/{a}^{2}}{\sqrt{1-{c}^{2}/{a}^{2}}})tan{h}^{-1}\sqrt{1-{c}^{2}/{a}^{2}}]$$

From these data, true area strain *ε* is calculated as shown in Equation (4), where the change in area *ΔA* is the difference between *A* and *A*_*o*_, the surface area of the uncompressed capsule.

4 $$\varepsilon =ln(1+\frac{{\rm{\Delta }}A}{{A}_{o}}).$$

**Figure 4:**
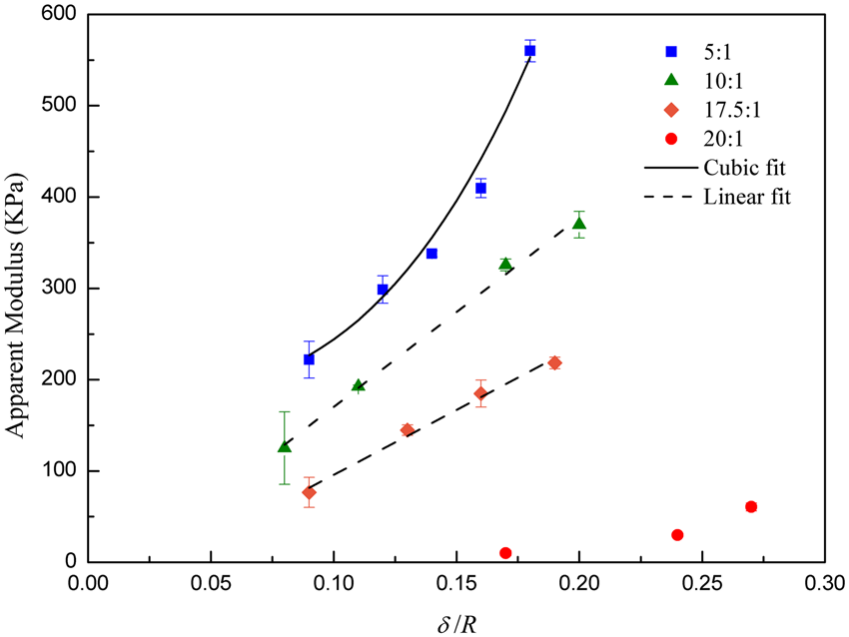
Plot of apparent modulus, defined as the ratio of the maximum stress to maximum strain in the compression tests, as a function of shell thickness (in units of capsule radius) for the different polymer to cross-link ratio tested. The continuous line represents a cubic fit for the 5:1 results. Dashed lines represent a linear fit for the 10:1 and 17.5:1 systems.

We evaluate the apparent modulus as the ratio of the maximum stress to the maximum strain and plot these values as a function of thickness, as shown in Fig. 4. Here, two trends are observed: one of a higher apparent modulus for higher cross-linked ratios, and the other of a higher modulus for larger δ*/R* values. In the former trend, a larger stress is required to deform the higher cross-linked ratio group (5:1), whereas the lowest stresses are required to deform the more flexible shells^20^ due to use of the lower-modulus bulk polymer^10^. This behavior is expected because the elastic modulus is directly proportional to the cross-link ratio^20,27^. Furthermore, the second trend observed is that within each cross-linker ratio system, the overall microcapsule stiffness increases with *δ*, thereby suggesting the shell thickness drastically influences the overall microcapsule stiffness^16,17^.

To further elucidate the effect of thickness within a given cross-linker ratio^5,16^ we curve-fit the data, as shown in Fig. 4. The bending stiffness for a membrane/shell is proportional to the cubic thickness while the stretching is proportional to the thickness^15^. The top curve representing the highest cross-linked ratio fits to a cubic power, thus indicating the stress proportionality to the cubic thickness; this relationship shows the bending or flexural rigidity is the predominant contributor to the overall stiffness of the capsules prepared with the 5:1 PDMS. By contrast, the 10:1 and 17.5:1 curves are linear, hence emphasizing the importance of the stretching response for the lower cross-linked ratios. Unexpectedly, at the lowest cross-link ratio of 20:1, the shells become too fragile to withstand significant stress, consequently preventing evident data trends. The fragility of the shells with the lowest cross-linking ratio suggests there is a critical limit for which the cross-link ratio becomes more significant to the overall stiffness than does the shell thickness. Moreover, when shells are thin^26,28^ bending is typically loosely defined and considered negligible. Consequently, the bending to stretching regime is solely defined as a thickness dependent condition. However, the differences between the curves in Fig. 4 for the various cross-linked densities indicate shell elasticity is also an important factor in the bending to stretching transition. A similar phenomenon is observed for thin blistering films^29^ in which a dimensionless parameter, *k*, is proportional to the square root of the radius squared over the modulus. At a fixed thickness, increasing the Young’s modulus lowers *k*. Consequently, the transition from bending to stretching is defined for low values of *k*, which corresponds to a higher stiffness. Regardless of the regime of deformation, all microcapsules will bend or stretch under stress; however, with the high cross-linked ratio, the stress is primarily attributed to bending, whereas for lower cross-linked ratio capsules, the stress is governed by stretching.

We hypothesize that the stiffer microcapsules will reduce fluid mobility to a larger extent than the softer microcapsules^27^. To evaluate this hypothesis, we flow the microcapsules through constrictions; these are prepared by using the capillary puller. A cylindrical glass capillary (with an inner diameter of 860 µm) is pulled to generate a constriction smaller than the average microcapsule diameter (*2R* = 707.5 µm). The constriction diameter of approximately 400 µm enables the microcapsules to deform to ~57% of their initial diameter. By coupling a pressure transducer to the capillary flow system, the pressure changes caused by microcapsules restricting fluid flow can be monitored. For each experiment, a syringe pump drives the microcapsule and its surrounding fluid (Milli-Q^®^ water) at a fixed volumetric flow rate through the constriction. Each capsule slowly and continuously deforms as it passes through the constriction; this behavior is characteristic of a lubrication flow between the PDMS and the glass surface. To evaluate the contribution of lubricated friction to the pressure drop, we obtain an estimate of *µ*_*f*_ = 0.01^30^. Thus, the friction force contributes only to approximately 4% of the total pressure drop acting on the capsule as it flows through the constriction.

**Figure 5:**
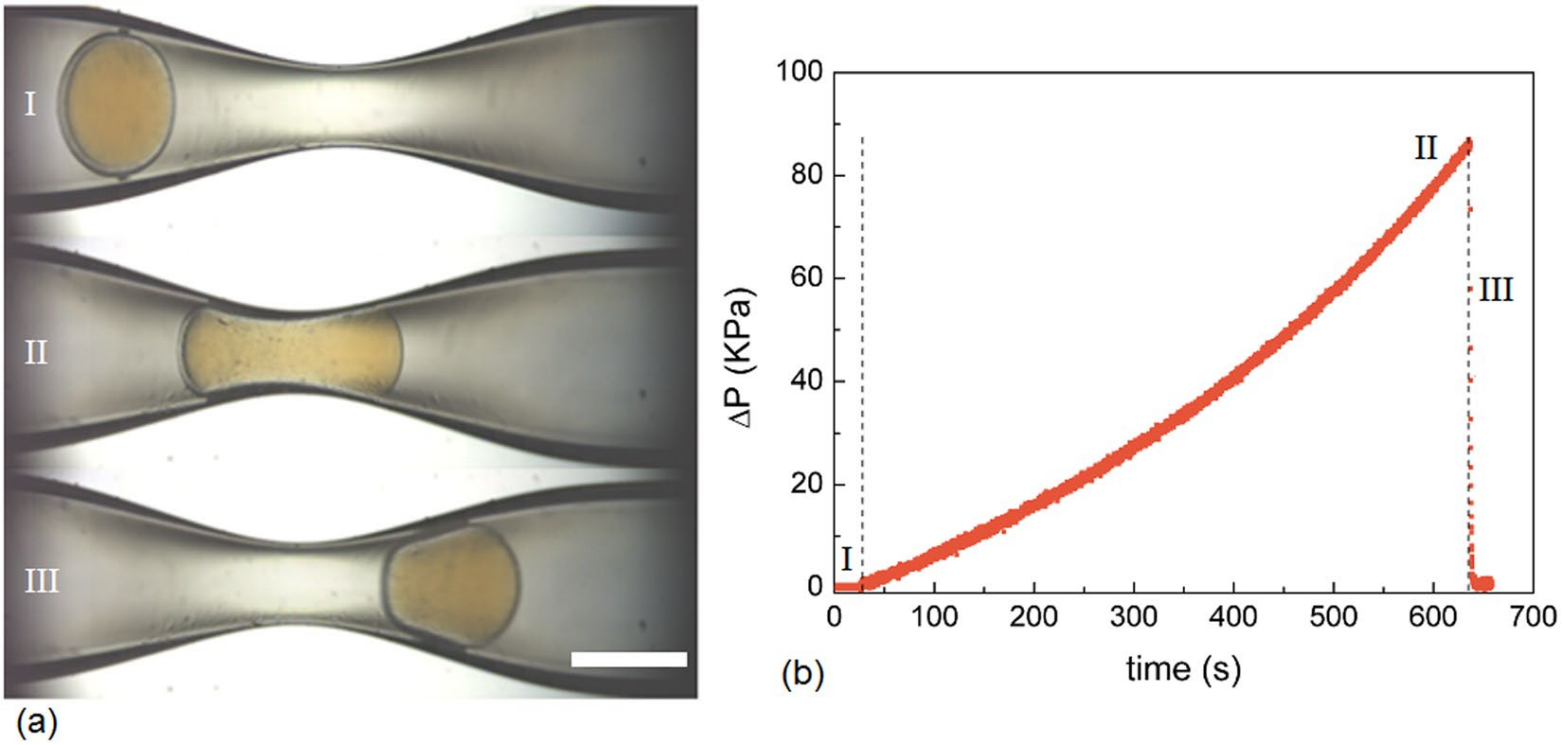
(**a**) Images of microcapsule (17.5:1, δ*/R* = 0.2) deformation as it flows through the constriction. The scale bar is 500 μm. (**b**) Evolution of the pressure-drop of the flow of a capsule and the suspending liquid (Milli-Q^®^ water) through a constriction.

The three positions (I, II, and III) of the microcapsule as it passes through the constriction are shown in Fig. 5a; these positions are directly correlated to the measured pressure difference (*ΔP*) as a function of time, shown in Fig. 5b. Each microcapsule position is defined by a distinct deformation regime: pre-deformation, maximum deformation, and recovery. The pre-deformation regime occurs when the microcapsule remains spherical prior to entering the constriction, as shown in Fig. 5a,I. In the pre-deformation regime, the position of the microcapsule is associated with the low-pressure region, shown in Fig. 5b,I; this pressure difference is equivalent to that of the suspending liquid alone. By contrast, a larger pressure is required to deform the capsule and push it through the constriction, as shown in Fig. 5b,II. As a result, in the maximum deformation state, the microcapsule deforms to a dumbbell shape thereby occupying the constriction and restricting fluid flow. The pressure increases to a maximum until the advancing microcapsule ceases to block fluid flow and it recovers to its initial spherical shape. The microcapsule then exits constriction into the wider opening of the unconstricted region of the capillary; this transition is coupled to an immediate drop in pressure, seen in Fig. 5b,III.

**Figure 6:**
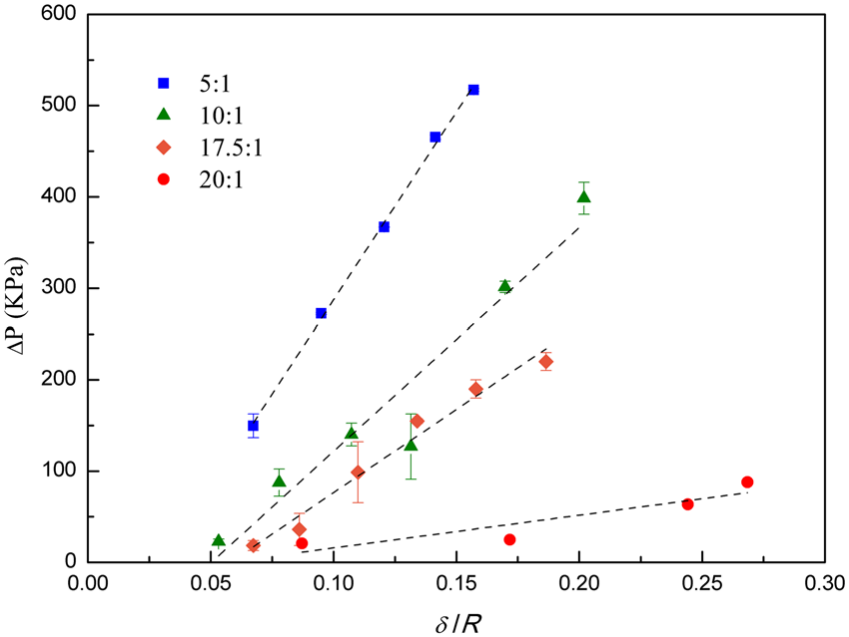
Maximum pressure-drop of the flow through a constriction as a function of shell thickness and polymer to cross-link ratio. The mobility reduction associated with the capsule passing through the constriction is a function of both the bulk modulus of the polymer (polymer to cross-link ratio) and capsule shell thickness.

We plot the maximum pressure difference as a function of shell thickness δ for each cross-link ratio to assess the impact of overall microcapsule stiffness on the ability of the microcapsule to change the mobility of the fluid flow, as shown in Fig. 6. As the thickness and stiffness increase, the resistance to fluid flow increases. For 5:1, the pressure increases with an approximate slope of ~5kPa (coefficient of determination = 0.998) and for the 20:1 increases with an approximate slope of 1kPa (coefficient of determination = 0.833). Moreover, the maximum pressure for the 5:1 group is six times the highest pressure from the 20:1 group because the microcapsules with high overall stiffness (more rigid) require more pressure to deform^21^. In addition, there is a trend of increased pressure with shell thickness, which is somewhat disrupted by an outlier in the 10:1 group. Nevertheless, at the highest thickness values for each cross-linking ratio, the pressure is largest for the high cross-linking ratio (5:1), and smallest at the low ratio (20:1), signaling the proportionality between the overall microcapsule stiffness and the ability of the microcapsule to increase fluid flow resistance. Thus, the extent of mobility control can be tuned by the elastic properties of the microcapsules suspended in a continuous phase.

## Conclusion

We controllably produced monodisperse microcapsules with tunable overall stiffness determined by two factors: the PDMS cross linking ratio that defines the polymer stiffness and the shell thickness. Single microcapsule compression tests show high cross-linking ratio shells require greater stress to deform. Moreover, the deformation mode of bending or stretching is determined by both the PDMS cross-linking ratio and the shell thickness, thus indicating the significance of both parameters in the design of elastically tunable microcapsules. Furthermore, the microcapsules with the high cross-linking ratio are able to reduce fluid mobility through a constricted passage to the greatest extent, whereas lower cross-linking ratios have a smaller range of effectiveness as mobility control agents in constricted media.

The strategies presented here enable the design of elastic capsules for use in confined environments with different flow behavior requirements. Suspensions of more rigid microcapsules can be used in porous media at which a high degree of mobility reduction of the water phase is needed, whereas softer microcapsules can be used in applications in which the intensity of the mobility reduction cannot be very high.

## Materials and Methods

The microcapsules are produced with the use of a double emulsion glass microcapillary device^24^. The device is composed of an outer square capillary (outer diameter, OD, 1.5 mm, inner diameter, ID, 1.05 mm, manufactured by Atlantic International Technology, Inc.), circular injection and collection capillaries (OD 0.58 mm, ID 1.00 mm, World Precision Instruments, Inc.), dispensing needles (type 304, McMaster-Carr) for fluid injection and a 25 mm × 75 mm glass microscope slide. The circular injection capillary is pulled with a micropipette puller (P-1000, Sutter Instrument) to a tapered opening of 40 µm and subsequently sanded to a diameter of ~150 µm. Then it was surface treated with Acquapel^®^ to make it hydrophobic. The capillaries are coaxially aligned manually with the aid of a microscope for visualization. The fluids are injected with the use of three syringe pumps (Harvard Apparatus 11 Elite). The inner fluid is Milli*-*Q^®^ (Millipore Corporation) water with food dye for visualization purposes, the middle fluid is the PDMS (Sylgard 184^®^ from Dow Corning base and curing agent). A 10% polyvinyl alcohol (PVA) solution is used in the outer phase to increase its viscosity and to serve as a surfactant. The production of the microcapsules is monitored with a high-speed camera (Photron SA3 Fast Camera) attached to an inverted microscope (Leica DMi8). Images are acquired at 1000 fps.

The mechanical characterization is performed with a Rheometer (DHR-3, TA Instruments) using a 40 mm diameter stainless steel plate-plate geometry. Each capsule is placed in 20 µL of water (enough liquid to cover the entire capsule). The procedure consists of several steps of compression where the axial force is recorded after steady state is reached at each condition. Compression is performed only up to a predetermined deformation in order to avoid capsule rupture.

A syringe pump is used to flow a single microcapsule suspended in Mili- Q^®^ water through a glass capillary (OD 1.5 mm, ID 0.86 mm, World Precision Instruments, Inc.) with a constriction, of inner diameter of approximately 0.40 mm. The capillary constriction is produced using a micropipette puller. For all tests, the pump is set to inject at a constant flow rate of 6 mL/h. A port, connected to a pressure transducer (Valydine) is installed to measure the pressure difference during the flow of the capsule through the constricted capillary. Pressure values are acquired on a PC by using a multiplexer slot as interface between the transducer and the computer. The inverted microscope and a high-speed camera are used to monitor and record the image of the microcapsule flowing through the capillary. For both the mechanical characterization and the microcapsule flow through a constriction, the results correspond to an average of at least three measurements.

## Electronic supplementary material

Supplementary Video 1
